# IgT Plays a Predominant Role in the Antibacterial Immunity of Rainbow Trout Olfactory Organs

**DOI:** 10.3389/fimmu.2020.583740

**Published:** 2020-11-16

**Authors:** Fen Dong, Guang-mei Yin, Kai-feng Meng, Hao-yue Xu, Xia Liu, Qing-chao Wang, Zhen Xu

**Affiliations:** ^1^Department of Aquatic Animal Medicine, College of Fisheries, Huazhong Agricultural University, Wuhan, China; ^2^Laboratory for Marine Biology and Biotechnology, Qingdao National Laboratory for Marine Science and Technology, Qingdao, China; ^3^Key Laboratory of Marine Biotechnology of Fujian Province, Institute of Oceanology, Fujian Agriculture and Forestry University, Fuzhou, China

**Keywords:** rainbow trout, immunoglobulins, B cells, nasopharynx-associated lymphoid tissue, bacterial infection

## Abstract

The olfactory organs (OOs) of vertebrates play important roles in their extraordinary chemosensory capacity, a process during which they are continuously exposed to environmental pathogens. Nasopharynx-associated lymphoid tissue (NALT) contains B cells and immunoglobulins (Igs), which function as the first defense line against antigens in mammals and also exist in teleosts. However, the immune responses of teleost NALT B cells and Igs during bacterial infection remain largely uncharacterized. In this study, rainbow trout were infected with *Flavobacterium columnare via* continuous immersion, after which the adaptive immune responses within NALT were evaluated. *F. columnare* could invade trout nasal mucosa and cause histopathological changes in trout OO. Moreover, the accumulation of IgT^+^ B cells in trout nasal mucosa was induced by bacterial challenge, which was accompanied by strong bacteria-specific IgT responses in the nasal mucus. Importantly, our study is the first to report local nasal-specific immune responses in teleosts during bacterial challenge by characterizing the local proliferation of IgT^+^ B cells and generation of bacteria-specific IgT in trout OOs after *F. columnare* infection. In addition to the strong IgT and IgT^+^ B cells responses in OO, bacteria-specific IgT and IgM were also detected in serum following bacterial challenge. Taken together, our findings suggest that IgT functions as an important mucosal Ig in teleost NALT and mediates local adaptive immunity during bacterial infection, which is similar to their protective role during parasitic infection.

## Introduction

The olfactory organs (OOs), which vertebrates rely on to identify substances in the environment, are continuously exposed to external microorganisms, and therefore a powerful defense system in their mucosal surface against infection is crucial (1). Particularly, teleost fish must actively draw water containing dissolved chemicals into their OOs to sense olfactory signals (2). At the same time, olfactory organs also experience continuous stimuli from the environments, which contain numerous toxins and pathogens. Therefore, a strong immune system is vital to protect the mucosal surface of OOs from pathogenic invasion. In birds and mammals, the nasopharynx-associated lymphoid tissue (NALT), which is composed of organized lymphoid tissue (O-NALT) (i.e., tonsils) and diffuse NALT (D-NALT), is generally considered the first line of defense against antigens (1, 3). However, only D-NALT is present in most teleosts and shares the main features of other teleost mucosa-associated lymphoid tissues (MALTs) (4). Teleost D-NALT has been found to contain abundant myeloid cells, T cells, and B cells (4). Moreover, in mammals, long-term virus-specific immune responses have been reported in the D-NALT but not in the O-NALT following exposure to the influenza virus, suggesting that D-NALT may play a key role in nasal antibody-mediated immunity (5).

Teleost fish represent the most ancient bony vertebrates that possess immunoglobulins (Igs). Unlike mammals, which express five Ig isotypes, only three Ig classes have been identified in teleosts (i.e., IgM, IgD, and IgT/IgZ) (6). IgM is the prevalent Ig class in teleost plasma, which is mainly responsible for systemic immunity (6, 7). IgD is also an ancient Ig class that has been identified in most teleost fish (8) and secreted IgD (sIgD) coats a lower percentage of microbiota in fish mucosal surfaces when compared to IgT or IgM (9, 10), and its function still requires further characterization. Additionally, teleost IgT (also known as IgZ) was the third Ig class described in 2005 (11, 12), which was demonstrated to mainly function in mucosal immunity, analogous to mammalian IgA (7). Upon parasitic challenge, IgT/IgZ was the dedicated Ig isotype in mucosal immune tissues including the gut, gills, and OO, whereas IgM was mainly involved in systemic immune responses (7, 9, 13). In mammals, bacteria-specific IgA is elevated for bacterial elimination in nasal mucus after pathogenic infection (14, 15), whereas the Ig-mediated adaptive immunity in the teleost OO during bacterial infection is not well understood. Given that teleost IgT shares a similar dedicated Ig role in mucosal tissues as mammalian IgA during parasitic infection (13), we hypothesized that sIgT in the teleost NALT is also a dedicated Ig isotype that mediates adaptive immunity upon bacterial infection.

*Flavobacterium columnare*, the causative pathogen for columnaris disease in many fish species including common carp (*Cyprinus carpio*), rainbow trout (*Oncorhynchus mykiss*), yellow perch (*Perca flavescens*), and whitefish (*Coregonus clupeaformis*) (16–19) is known to cause severe mucosal tissue damage and high mortalities in infected fish (19, 20). However, the role of Ig-mediated adaptive immunity in fish NALT during bacterial infection remains unknown. In this study, we found that *F. columnare* could invade the trout OO to induce severe histological changes and IgT in nasal mucus played a central role against bacterial challenge. Moreover, we identified the local proliferation of IgT^+^ B cells in nasal mucosa and bacteria-specific IgT responses in nasal mucus against bacterial infection for the first time in teleosts. Therefore, our results highlight the predominant role of sIgT in teleost NALT against bacterial infection.

## Materials and Methods

### Fish Maintenance

Rainbow trout (mean weight, 3–5 g) used in the study were obtained from Gerui fish farm in Shiyan (Hubei, China), and maintained in the aquarium tanks using a water recirculation system involving thermostatic temperature control and extensive biofiltration. Fish were acclimatized for at least 14 days at 16°C and fed daily with commercial trout pellets at a rate of 0.5%–1% body weight during the whole experiment period. The feeding was terminated at 48 h before sampling. For sampling, fish were anesthetized with MS-222 and blood was removed through the caudal vein to minimize the blood content in the collected organs. Animal procedures were approved by the Animal Experiment Committee of Huazhong Agricultural University.

### *F. columnare* Strain and Infection

The bacteria used in this study was *Flavobacterium columnare* G_4_ strain, obtained from professor Pin Nie’s lab in the Institute of hydrobiology, Chinese academy of sciences. *F. columnare* strain G_4_ was cultured in Shieh broth as described previously (21). Total 30 Fish (~ 3–5 g) were immersed with *F. columnare* at a final concentration of 1 × 10^6^ CFU ml^–1^ for 4 h at 16°C, and then transferred into the new aquarium. The olfactory organ samples were collected at 1, 2, 4, 7, 14, 21, 28, and 75 days post infection (dpi). Moreover, fluids (serum and nasal mucus) were taken after 28 days, which was set as the infected group. For prolonged immune response analysis, fish were exposed to bacteria twice again at 30^th^ day and 60^th^ day after the first infection with the same bacterial dosage. Fish samples were taken two weeks after the last challenge, i.e. 75^th^ day post first infection, which was set as the survivor group. As a control (mock infected), the same number of fish were maintained in a similar tank with the same culture medium without bacteria. To explore the invasion route and tissue distribution of bacteria in trout OO, we used the special GFP-expressing *F. columnare* G_4_ strain (green fluorescent protein (GFP)-labeled mutant which is stable inheritance and expression) to infect rainbow trout *via* immersion method as above. The olfactory organ samples were collected at 1, 2, 3, 4, and 7 dpi.

### Histology, Light Microscopy, and Immunofluorescence Microscopy Studies

The olfactory organ from the control trout and infected trout were dissected and fixed with 4% neutral buffered formalin at 4°C overnight, then embedded in paraffin, and 5 μm thick sections for hematoxylin and eosin (H&E) or Alcian blue (A&B) staining as reported previously (13). Images were acquired in a microscope (Olympus) using the Axiovision software.

To detect the localization of *F. columnare* in trout olfactory organ, we used GFP-expressing *F. columnare* for infection. All the sections were stained with DAPI (4’, 6-diamidino-2-phenylindole; 1 μg ml^–1^; Invitrogen). In order to detect the IgT^+^ and IgM^+^ B-cells, polyclonal rabbit anti-trout IgT (pAb; 0.5 μg ml^–1^) and monoclonal mouse anti-trout IgM (IgG1 isotype; 1 μg ml^–1^) (7) were used to incubate sections at 4°C overnight. After washing three times with PBS, secondary antibodies Alexa Fluor 488-conjugated AffiniPure Goat Anti-Rabbit IgG (H+L) and Cy3-conjugated AffiniPure Goat Anti-Mouse IgG (H+L) (2.5μg ml^–1^ each; Jackson ImmunoResearch Laboratories Inc.) were added and incubated for 40 min at room temperature. Then after washing three times with PBS, sections were stained with DAPI (4’, 6-diamidino-2 phenylindole; 1 μg ml^-1^; Invitrogen) for 8 min. All the sections were mounted with antifade mounting medium (Beyotime). Images were acquired and analyzed using an Olympus BX53 fluorescence microscope (Olympus) and the iVision-Mac scientific imaging processing software (Olympus).

### RNA Isolation and Quantitative Real-Time PCR Analysis

Total RNA was isolated using the TRIZol reagent according to the manufacturer’s instructions. All the samples for quantitative real-time PCR (qPCR) analyses were homogenized by TissueLyser II (Jingxin Technology) with steel beads at shaking (60 Hz for 1 min) following the manufacturer’s instructions. The concentrations of the isolated RNA were carried out by NanoDropND-1000 spectrophotometer (Thermo Scientific), and the integrity of tissue RNA was determined by agarose gel electrophoresis. To normalize gene expression levels, equivalent amounts of the total RNA (1 µg) from each sample was used for cDNA synthesis. The cDNA synthesis was performed the SuperScript first-strand synthesis system (Yeasen) in a 20 μl reaction volume. The qPCRs were performed on a 7500 qPCR system (Applied Biosystems) using the 2× SYBR Green qPCR Master mix (Yeasen). The synthesized cDNA was diluted 4 times and then used as a template for qPCR analysis. All samples were performed the following conditions: 95°C for 5 min, followed by 40 cycles at 95 °C for 10 s and at 58°C for 30 s. A dissociation protocol was carried out after thermos cycling to confirm that a single band of the correct size was amplified. Ct values determined for each sample were normalized against the values for trout housekeeping gene (EF1α). The gene expression levels were shown as 2^–ΔΔCt^ using the Pfaffl’s method (22). Primer sequences (species-specific 16S rRNA primers were used for *F. columnare*) can be found in **Table 1**.

**Table 1:** Primers used in this study.

Gene	Primer name	Primer sequence (5´-3´)	GenBankAccession no.
16S rRNA	Fc16F	GAGTGGCTAAGCGAAAGTGAT	EU395796.1
	Fc16R	ACCTGACACCTCACGGCAC	
C1R	C1RF	GCCGACTGCTACTACGA	NM_001124380.1
	C1RR	GGTGCCTATGTGCTCTATG	
IL-8	IL-8F	TGTCGTTGTGCTCCTGG	NM_001124362.1
	IL-8R	CCTGACCGCTCTTGCTC	
TNF-α	TNF-αF	AACCGAAAGTGCGAGTG	XM_021565683.1
	TNF-αR	TGGTGCGATCTGGAGTA	
HSPA1s	HSPA1sF	GATGGACAAGGCTCAGGT	XM_021567143.1
	HSPA1sR	CGTCAAGGAGGAGAAGGT	
NOS2	NOS2F	GGCAGTCAAGAACCAACC	XM_021581479.1
	NOS2R	GAGCACCAAACGCTAATT	
IgD	IgDF	CAGGAGGAAAGTTCGGCATCA	AY748802.1
	IgDR	CCTCAAGGAGCTCTGGTTTGGA	
IL-10	IL-10F	CACCGCCTTCTCCACCATC	NM_001245099.1
	IL-10R	CCATAGCGTGACACCCCAC	
CATH-1	CATH-1F	CTGGAGGCAAGCAACAAC	NM_001124480.1
	CATH-1R	CCCCCAAGACGAGAGACA	
IgM	IgM F	AAGAAAGCCTACAAGAGGGAGA	S63348.1
	IgM R	CGTCAACAAGCCAAGCCACTA	
IgT	IgTF	CAGACAACAGCACCTCACCTA	AY870264
	IgTR	GAGTCAATAAGAAGACACAACGA	
IRF4	IRF4F	CGCCCTACGGAGATAAAC	XM_021604510.1
	IRF4R	TCAGCAGCACCTGGAGAC	
CXCL10	CXCL10F	ACATCAACGGTCCTCATC	XM_021622222.1
	CXCL10R	ACACTTCTTCCCTTCTCC	
C3	C3F	CCTCACAACAAGAGTGCACATC	XM_021561577.1
	C3R	CCAAGTGGGCAAACTCATCTCC	
SAA	SAAF	TTGTTCTGACCCTCGTTG	XM_021607573.1
	SAAR	CCTGGCAGCATCATAGTT	
pIgR	pIgRF	GTACAGCAGGTGTTCACAGTAAC	FJ940682.1
	pIgRR	CCACAGACAGACCTTGGATAAC	
EF1α	EF1αF	CAACGATATCCGTCGTGGCA	NM_001124339.1
	EF1αR	ACAGCGAAACGACCAAGAGG	

### DNA Extraction and PCR Amplification

To detect *F. columnare* in trout nasal after infection, the homogenates of olfactory organ in the control fish and infected fish were cultured in the plate, using steel beads and shaking (60 Hz for 1 min) following the manufacturer’s instructions. One day later, the bacteria in the plate of infected fish were collected, and those colonies present rhizoid and flat with yellow center were then diluted in the Shieh broth for 24 h culture at 28°C. PCR reactions were performed in triplicate 20 μl mixtures containing 4 μl of 5 × FastPfu Buffer, 2 μl of 2.5 mM dNTPs, 0.8 μl of each primer (5 μM)(F1: 5’-GCCCAGAGAAATTTGGAT-3’ R1: 5’-TGCGATTACTAGCGAATCC -3’) (23), 0.4 μl of FastPfu Polymerase and 12 μl bacteria solution. The PCR reactions were conducted using the following program: 3 min of denaturation at 95°C, 27 cycles of 30 s at 95°C, 30 s for annealing at 55°C, and 45 s for elongation at 72°C, and a final extension at 72°C for 10 min. Agarose gels were used to exhibit the PCR products and image was acquired from the ChemiDoc TM XRS+ imaging system (Bio-Rad).

### Collection of Serum and Nasal Mucus

Before sampling, trout were anesthetized with MS-222, and serum was collected and stored as previously described (7). The nasal mucus samples were collected using the method described previously (13). Briefly, trout olfactory organ was collected (together with bones surrounding the nasal cavity) and then washed three times with PBS on a shaker (each time 10 min) to remove the remaining blood in the tissue and the mucus in the tissue’s surface. The nasal cavity was exposed *via* cut with scissors and was incubated for 12 h at 4°C, with slightly shaking in protease inhibitor buffer (1× PBS, containing 1× protease inhibitor cocktail [Roche], 1 mM phenylmethylsulfony fluoride [Sigma]; pH 7.2) at a ratio of 100 mg of the tissue per ml of buffer. The suspension (nasal mucus) was collected into 1.5 ml centrifuge tube, and then vigorously vortexed and centrifuged at 400 g for 10 min at 4°C to remove trout cells. The cell-free supernatant was thereafter centrifuged at 10,000 g for 10 min at 4°C to remove the nasal bacteria from mucus. Finally, the resulting supernatant (containing nasal mucus) was harvested, filtered with a 0.45 μm syringe filter (Millipore) and stored at 4°C prior to use the same day.

### SDS-PAGE and Western Blot

Nasal mucus and serum samples were resolved on 4%–15% SDS-PAGE Ready Gel (Bio-Rad) under non-reducing conditions as described previously (9, 13). For western blot analysis, the proteins on the gels were transferred onto PVDF membranes (Bio-Rad). Then, the membranes were blocked with 8% skim milk and incubated with anti-trout IgT (rabbit polyclonal antibody, pAb), anti-trout IgM (mouse monoclonal antibody, mAb) or biotinylated anti-trout IgD (mouse mAb) (9) antibodies followed by incubating with peroxidase-conjugated anti-rabbit, anti-mouse IgG (Invitrogen) or streptavidin (Invitrogen). For quantitative analyses of IgT, IgM, and IgD in nasal mucus and serum, immunoreactivity bands were first visualized with an enhanced chemiluminescent reagent (Advansta) and scanned by Amersham Imager 600 Imaging System (GE Healthcare), then band densitometry was analyzed with ImageQuant TL software (GE Healthcare). Finally, the concentrations of IgT, IgM, and IgD were determined by plotting the obtained signal strength values on a standard curve generated for each blot using known amounts of purified trout IgT, IgM, or IgD.

### Proliferation of B Cells in the Olfactory Organ of Trout

The proliferation of B cells was assayed using the revised methodology as previously reported (9, 13, 24). Briefly, both the control fish and survivor fish (~ 15 g) were anesthetized with MS-222 and intravenously injected with 200 μg 5-ethynyl-2’-deoxyuridine (EdU) (Invitrogen), and 24 h later, the olfactory organ from control and survival fish were dissected, fixed in 4% neutral buffered formalin as described above. Paraffin sections were incubated with polyclonal rabbit anti-trout IgT (pAb; 0.5 μg ml^–1^) and monoclonal mouse anti-trout IgM (IgG1 isotype; 1 μg ml^–1^) at 4˚C overnight. After washing three times with PBS, sections were incubated with Alexa Fluor 488-conjugated AffiniPure Goat anti-rabbit IgG and Cy3-conjugated AffiniPure Goat anti-mouse IgG (2.5 μg ml^–1^ each; Jackson ImmunoResearch Laboratories Inc.) for 45 min at room temperature. For the detection of EDU^+^ cells, we used the kit according to the manufacturer’s instructions (Click-iT EdU Alexa Fluor 647 Imaging Kit, Invitrogen). The nuclei were stained with DAPI (4’, 6-diamidino-2 phenylindole; 1 μg ml^-1^: Invitrogen) for 8 min. Images were acquired and analyzed using an Olympus BX53 fluorescence microscope (Olympus) and the iVision-Mac scientific imaging processing software (Olympus). The percentage of EdU^+^Ig^+^ positive cells means the ratio of EdU^+^Ig^+^ cells to Ig^+^ cells. The percentage of EdU^+^ cells means the ratio of EdU^+^ cells to trout OO cells.

### Tissue Explants Culture

The culture of trout olfactory organ explants was conducted using the similar method as previously described (9, 13). Briefly, both control and survivor fish were sacrificed with an overdose of MS-222, and blood was removed through the caudal vein to minimize the blood content in the collected organs. Thereafter, olfactory organ (approximately 20 mg of each tissue) was submerged in 70% ethanol for 30 s to eliminate possible bacteria on their surface and then washed twice with PBS. Finally, tissues were placed in a 24-well plate and cultured with 120 μl DMEM medium (Invitrogen), supplemented with 10% FBS, 100 U ml^–1^ penicillin, 100 μg ml^–1^ streptomycin, 200 μg ml^–1^ amphotericin B and 250 μg ml^–1^ gentamycin sulfate, with 5% CO2 at 17˚C. After 7 days culture, the supernatants were harvested, centrifuged and stored at 4°C prior to use the same day, otherwise, stored at −80°C until further analysis.

### Binding of Trout Immunoglobulins to *F. columnare*

To access whether the infected fish and survivor fish generated F. columnare-specific immunoglobulins, the capacity of IgT, IgM and IgD from serum, nasal mucus or tissue of olfactory organ explants supernatants binding to *F. columnare* was assayed using a pull-down assay as described previously (9, 13). Briefly, the *F. columnare* suspensions (1×108 CFU ml^–1^) were pre-incubated with a solution of 0.5% BSA in PBS (pH 7.2) at 4°C for 2 h. Thereafter, *F. columnare* (40 μl) suspensions were incubated with diluted nasal mucus, serum or tissue of olfactory organ explants supernatants from the infected fish, survivor fish or control fish at 4°C for 4 h with continuous shaking in a 300 μl volume with PBS containing 1% BSA (pH 7.2). After incubation, the bacteria were washed three times with PBS, and bound proteins were eluted with 2×Laemmli Sample Buffer (Bio- Rad) and boiled for 5 min at 95°C. The eluted material was resolved on 4–15% SDS-PAGE Ready Gel (Bio-Rad) under non-reducing conditions, then the presence of IgT, IgM or IgD was detected by western blotting using the anti-trout IgT, IgM, or IgD antibody as described above.

### Statistics

An unpaired Student’s t-test and one-way ANOVA with Bonferroni correction (Prism version 6.01; GraphPad) were used for analysis of differences between two groups or among three or more groups, respectively. Data were expressed as mean ± SEM. All statistical significance in this study was set at *P* < 0.05.

## Results

### Detection of *F. columnare* in Trout OO After Infection

**Figure 1:**
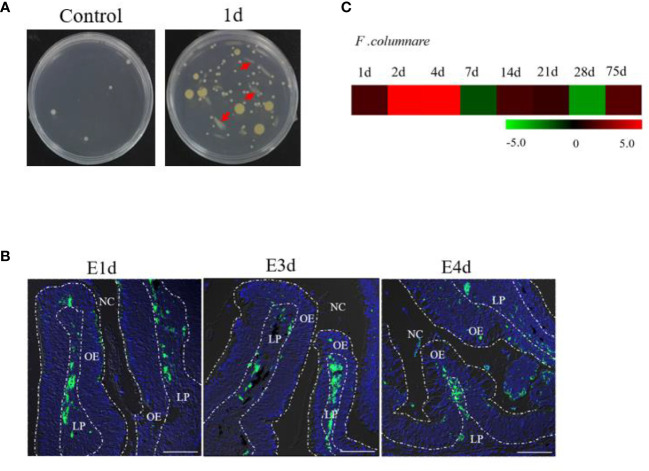
Infection with *F. columnare* in rainbow trout. **(A)** The culture plates of olfactory organ from control fish (left) and 1 day infected fish (right). **(B)** localzation of *F. cloumnare* in olfactory organ of control fish and infected fish at 1, 3, and 4 dpi. Data are representative of three independent experiments. **(C)** Heat map demonstrated quantitative real-time PCR (qPCR) results of *F. columnare* in olfactory organ of infected fish versus control fish measured at 1, 2, 4, 7, 14, 21, 28, and 75 dpi (n = 6 fish per group). NC, nasal cavity; OE, olfactory epithelium; LP, lamina propria. Scale bar, 100 μm.

Obvious clinical signs of columnaris disease (e.g., frayed fins and necrotic skin lesions) were detected in *F. columnare*-infected rainbow trout (**Supplementary Figure 1A**). After incubating OO tissue homogenates in Shieh agar plates, yellow-pigmented rhizoid colonies (i.e., resembling *F. columnare* colonies) could be observed in cultures containing infected fish homogenates, whereas no such colonies were observed in control fish homogenate cultures (**Figure 1A**). Moreover, PCR cloning of three different bacterial colonies that present typical *F. columnare* characteristics exhibited bands that were similar to that of the positive control (**Supplementary Figure 1B**). To further explore the invasion route and tissue distribution of bacteria in trout OO, one special GFP-expressing *F. columnare* G_4_ strain was used to infect rainbow trout *via* immersion. Trout OO lamellae were found to harbor numerous green fluorescent bacteria at 1, 3, and 4 days post-infection (dpi) (**Figure 1B**). Quantitative real-time PCR (qPCR) analysis also confirmed the expression of *F. columnare* 16S rRNA in trout OO after bacterial infection (**Figure 1C**).

### Bacterial Infection Elicits Histopathological Changes and Immune Responses in Trout OO

**Figure 2:**
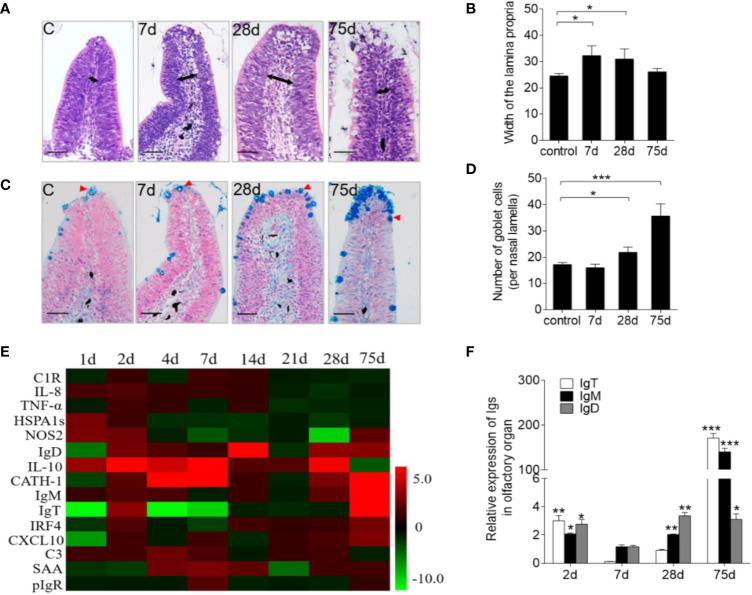
Pathological changes and kinetics of the immune responses in the olfactory organ of trout infected with *F. columnare*. **(A)** Histological examination by H & E staining of olfactory organ from uninfected fish and *F. columnare*-infected rainbow trout at 7, 28 and 75 dpi. Black arrows indicate the width of LP at the tip region (100 μm from the lamellar tip) of the olfactory lamella. **(B)** The width of LP at the tip region the olfactory lamella in control fish and *F. columnare* -infected rainbow trout at 7, 28, 75 dpi counted from A. **(C)** Histological examination by A & B staining of olfactory organ from uninfected fish and *F. columnare*-infected rainbow trout at 7, 28 and 75 dpi. Red arrowheads indicate goblet cells. **(D)** The number of goblet cells at the olfactory lamella in control fish and *F. columnare* -infected rainbow trout at 7, 28 and 75 dpi counted from C. **(E)** Heat map illustrates quantitative real-time PCR results of relative mRNA levels for selected immune markers in *F. columnare*-infected fish versus control fish measured at 1, 2, 4, 7, 14, 21, 28 and 75 dpi (*n* = 6 per group) in the olfactory organ of rainbow trout. Data are expressed as mean fold increase in expression. **(F)** The expression levels of IgM, IgD and IgT at 2, 7, 28 and 75 dpi with *F. columnare* in olfactory organ of rainbow trout. (*n* = 6 fish per group). LP: lamina propria. Scale bars, 50 μm. *p < 0.05, **p < 0.01, ***p < 0.001 (one-way ANOVA with Bonferroni correction). Data are representative of three independent experiments (mean ± SEM).

In addition to *F. columnare* detection, our study sought to characterize the associated morphological changes and immune-related gene expression in trout OO. Hematoxylin and eosin (H&E) staining showed a significant enlargement of the lamina propria (LP) at the tip of the nasal lamella (~ 100 μm away from the apex) in the infected fish when compared with that in the control fish (**Figures 2A, B**). A&B staining also demonstrated that the number of goblet cells on the nasal lamella increased significantly upon infection (**Figures 2C, D**). Moreover, the mRNA expressions of immune-related genes in trout OO were significantly up-regulated as early as 1 dpi (HSPA1s, NOS2, and IL-10) or 2 dpi (CATH-1 and IL-10) and reached their peak levels at 7 dpi (**Figure 2E**). Moreover, the transcriptional levels of IgT, IgM, and IgD were significantly increased at 2 dpi and reached their peak at 28 dpi (IgD) and 75 dpi (IgT and IgM) (**Figure 2F**).

### Bacterial Infection Elicits B Cell- and Igs-Mediated Adaptive Immune Response in Trout OO

**Figure 3:**
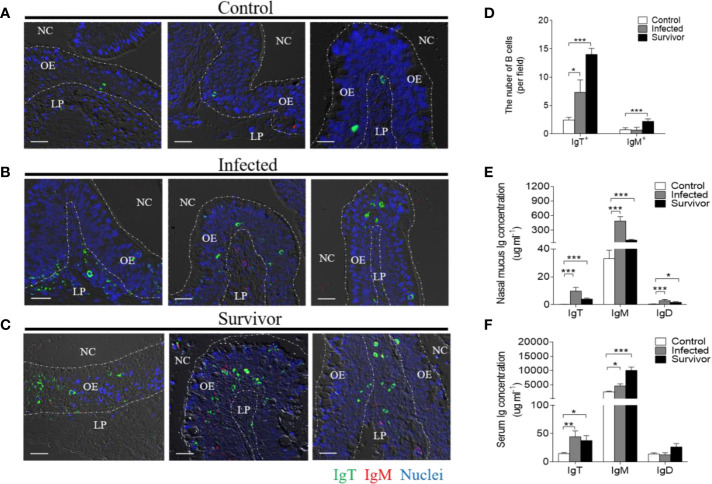
Responses of IgT^+^ B cells and Igs in the olfactory organ of trout infected with *F. columnare*. **(A–C)** Differential interference contrast images of immunefluorescence staining on paraffinic sections of olfactory organ from control fish **(A)**, infected fish (28 dpi, **B**) and survivor fish (75dpi, **C**). IgT^+^ and IgM^+^ B cells were stained with rabbit anti-trout IgT (green) and mouse anti-trout IgM (red), respectively; nuclei were stained with DAPI (blue) (isotype-matched control antibody staining, **Supplemental Figure 2**). **(D)** The number of IgT^+^ and IgM^+^ B cells in paraffinic sections of olfactory organ from control fish, infected fish (28 dpi), and survivor fish (75 dpi) (*n* = 6 fish per group). **(E, F)** Concentration of IgT, IgM, and IgD in nasal mucus **(E)** and serum **(F)** of control, infected (28 dpi), and survivor fish (75 dpi) (*n* = 12 per group). NC, nasal cavity; OE, olfactory epithelium; LP, lamina propria. Scale bar, 20 μm. *p < 0.05, **p < 0.01, ***p < 0.001 (one-way ANOVA with Bonferroni correction). Data in **(D–F)** are representative of at least three independent experiments (mean ± SEM).

**Figure 4:**
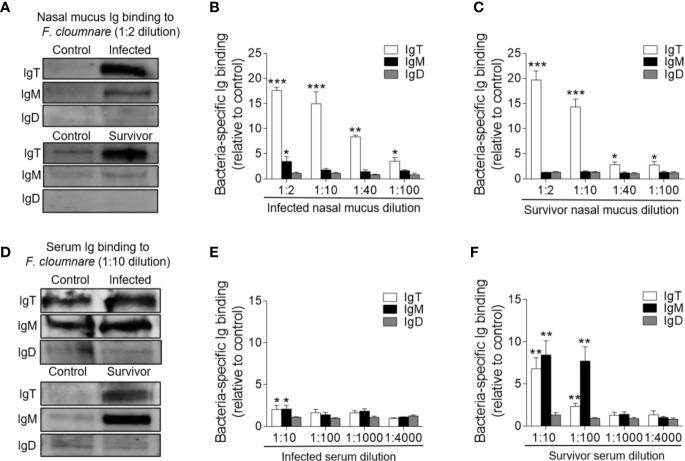
Immunoglobulin responses in the olfactory organ and serum from infected and survivor trout. **(A)** Western blot analysis of IgT-, IgM-, and IgD- specific binding to *F. columnare* in nasal mucus (dilution 1:2) from infected and survivor fish. **(B, C)** IgT-, IgM-, and IgD- specific binding to *F. columnare* in dilutions of nasal mucus from infected **(B)** and survivor fish **(C)**, evaluated by densitometric analysis of immunoblots and presented as relative values to those of control fish (*n* = 12 per group). **(D)** Western blot analysis of IgT-, IgM-, and IgD- specific binding to *F. columnare* in serum (dilution 1:10) from infected and survivor fish. **(E, F)** IgT-, IgM-, and IgD- specific binding to *F. columnare* in dilutions of serum from infected **(E)** and survivor **(F)** fish, evaluated by densitometric analysis of immunoblots and presented as relative values to those of control fish (*n* = 12 per group). *p < 0.05, **p < 0.01, ***p < 0.001 (unpaired Student *t* test). Data are representative of three independent experiments (mean ± SEM).

Immunofluorescence analysis was used to detect the abundance of IgT^+^ and IgM^+^ B cells in trout OO to demonstrate the role of B cells in the adaptive immune response in trout OO during *F. columnare* infection. Few IgT^+^ and IgM^+^ B cells were detected in both the olfactory epithelium and the lamina propria of control fish (**Figure 3A**; isotype-matched control antibodies, **Supplementary Figure 2**), whereas a moderate increase in IgT^+^ B cells was observed in the trout OO at 28 dpi (**Figure 3B**). Moreover, the number of IgT^+^ B cells was dramatically higher in surviving fish at 75 dpi compared to the controls (**Figure 3C**). Similarly, the number of IgM^+^ B cells in the trout OO did not significantly change in the infected fish (28 dpi) but significantly increased in surviving fish (75 dpi) compared to the controls (**Figures 3A–C**). The number of IgT^+^ B cells increased significantly by ~3 fold and ~6 fold in the OOs of infected and surviving fish, respectively, whereas the number of IgM^+^ B cells increased ~3 fold in the surviving fish (**Figure 3D**). Moreover, western blotting analyses demonstrated that IgT concentration in the nasal mucus of the infected and surviving fish increased by ~ 35 and ~ 15-fold of that in the control fish, respectively (**Figure 3E**). Notably, ~15- fold and ~3- fold increases of IgM concentrations were detected in the nasal mucus of infected and surviving fish, respectively, whereas IgD concentrations in the nasal mucus of the infected and survivor groups increased by ~ 6-fold and ~ 3-fold, respectively (**Figure 3E**). The serum concentrations of IgM and IgT increased in both the infected (~2 and ~3-fold, respectively) and surviving fish (~4 and ~3-fold, respectively) (**Figure 3F**), whereas serum IgD concentrations did not significantly change in either the infected or surviving fish (**Figure 3F**). Pull-down assays were then conducted to detect bacteria-specific Igs in nasal mucus following *F. columnare* infection (**Figure 4**). The results demonstrated that bacteria-specific IgT binding could be detected in up to 1/100 nasal mucus dilutions in both the infected and surviving fish, representing a ~3.5-fold and ~2.7-fold increase compared to the control, respectively (**Figures 4A–C**). Bacteria-specific IgM binding in serum could be detected in up to 1/10 and 1/100 dilutions in the infected and surviving fish, which were ~ 2-fold and ~ 7.7- fold higher than the control fish, respectively (**Figures 4D–F**). Similarly, bacteria-specific IgT binding could be detected in up to 1/100 serum dilutions of surviving fish, which represented a ~2.3-fold increase compared to the control fish but was still much lower than the bacteria-specific IgM (**Figure 4F**). No significant changes in the IgD titers in the nasal mucus or serum in infected and surviving fish were identified (**Figure 4**). These results suggested that Igs play an important role in adaptive immune responses in the trout OO upon *F. columnare* infection.

### Bacterial Infection Elicits Local Proliferation of B-Cells and Ig Responses in Trout OO

**Figure 5:**
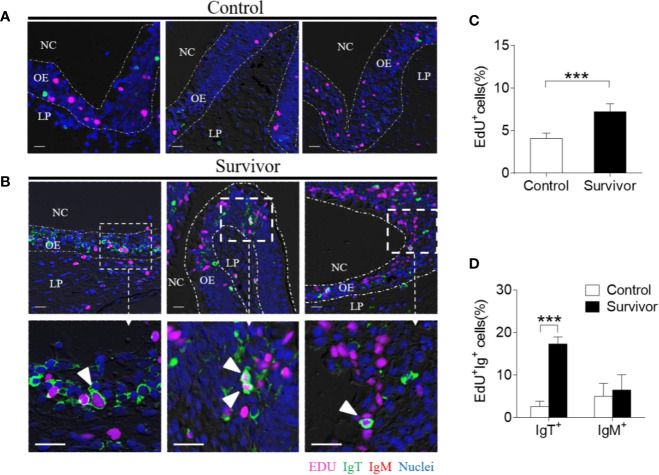
Proliferation of IgT^+^ B cells in the olfactory organ from survivor fish. **(A, B)** Immunofluorescence analysis of 5-ethynyl-2’-deoxyuridine (EdU) incorporation by IgT^+^ or IgM^+^ B cells in the olfactory organ of control **(A)** and survivor fish **(B)** and enlarged images of the areas outlined. Paraffinic sections of olfactory organ were stained for EdU (magenta), trout IgT (green), trout IgM (red), and nuclei (blue) detection. White arrowheads point to cells double stained for EdU and IgT. NC, nasal cavity; OE, olfactory epithelium; LP, lamina propria. Scale bars, 20 μm. **(C)** Percentage of EdU^+^ cells from total olfactory organ cells in control and survivor fish counted from A and B (*n* = 6 fish per group). We have counted 18 fields or 19 fields in the control or survivor group, respectively. **(D)** Percentage of EdU^+^ cells from the total olfactory organ IgT^+^ and IgM^+^ B cell populations in control and survivor fish, statistically calculated from A and B. ***p < 0.001 (unpaired Student *t* test). Data in **(C, D)** are representative of at least three independent experiments (mean ± SEM).

**Figure 6:**
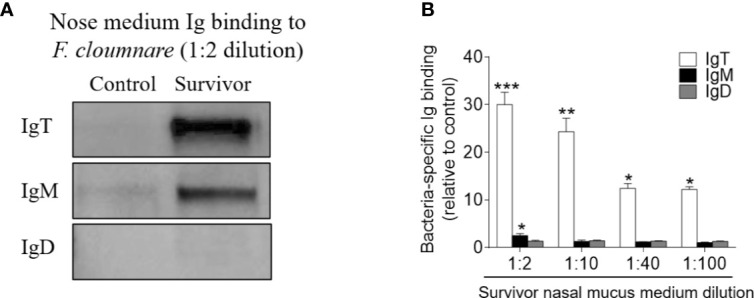
Local IgT-, IgM-, and IgD-specific responses in olfactory organ explants of survivor fish. **(A)** The olfactory organ explants from control and survivor fish were cultured in medium (400 μl) for 7 days. Western blot analysis of IgT-, IgM-, and IgD-specific binding to *F. columnare* in the olfactory organ culture medium (dilution 1:2) from control and survivor fish. **(B)** IgT-, IgM-, and IgD-specific binding to *F. columnare* in dilutions of olfactory organ culture medium from control and survivor fish, evaluated by densitometric analysis of immunoblots and presented as relative values to those of control fish (*n* = 12 per group). *p < 0.05, **p < 0.01, ***p < 0.001 (unpaired Student’s *t*-test). Data are representative of at least three independent experiments (mean ± SEM).

To investigate whether the increases in IgT^+^ B cells in the OO of surviving fish resulted from local proliferation, the *in vivo* proliferative responses of IgT^+^ and IgM^+^ B cells were evaluated by staining with 5-Ethynyl-2´-deoxyuridine (EdU), a thymidine analog that incorporates to DNA during cell division (25). Immunofluorescence microscopy analysis indicated that the percentage of proliferating cells in the OO of surviving fish (~ 7.22 ± 0.63%) significantly increased compared to the control fish (~ 4.07 ± 0.95%) (**Figures 5A–C**). Moreover, the percentage of EdU^+^IgT^+^ B cells also increased significantly in the surviving fish (~ 17.32 ± 1.63%) compared to the controls (~ 2.62 ± 1.26%) (**Figures 5A, B, D**), whereas no significant changes were found in the percentage of EdU^+^IgM^+^ B cells in trout OO between the control and surviving fish (**Figures 5A, B, D**). Bacteria-specific Igs titers were further measured in media containing cultured olfactory organs from the control and surviving fish (**Figure 6**). Bacteria-specific IgT binding could be detected in 1/100 diluted medium (~ 12-fold) containing cultured OO explants from the surviving fish, whereas much lower bacteria-specific IgM titers were detected in the same medium (**Figure 6**). Moreover, negligible bacteria-specific IgD titers were detected in the cultured olfactory organ media from either the control or surviving fish (**Figure 6**).

## Discussion

In mammals, NALT is considered within the first line of immune defense against inhaled antigens, a process during which IgA plays a central role (1, 5). Teleost fish represent the most ancient bony vertebrates with NALT and are known to express three Ig classes (4, 6), which mediate the immune defense mechanisms in the OO (4). However, very little is known about the role of Igs in nasal immune defense against bacterial pathogens. Therefore, our study explored the immune responses of Igs and B cells in teleost OO against a bacterial pathogen and identified IgT as the predominant Ig isotype in teleost NALT during antibacterial immunity.

Columnaris disease, which is caused by *F. columnare*, is known to induce histological lesions in multiple mucosal tissues of fish including rainbow trout (18). Here, GFP-expressing *F. columnare* was observed in the trout OO at 1 dpi, particularly in the lamina propria (LP), and typical clinical signs (e.g., frayed fins and necrotic skin lesions) were also observed in infected trout. Alcian blue is a cationic dye used to stain acidic polysaccharides, including bacterial capsules. At a pH of 2.5, this dye stains both sulfated (sulfomucins) and carboxylated (sialomucins) mucopolysaccharides, which are found in the goblet cells located in the intestine. Moreover, we found that GFP-expressing *F. columnar*e were mainly located in the lamina propria of the trout OO after challenge. Alcian blue was then used to detect goblet cells in the trout OO and only the cells located in the trout nasal epidermal layer were counted. Histological analyses indicated that the microscopic structure of the trout NALT significantly changed in infected fish, as both the width of the LP and goblet cell density in the lamellae significantly increased in the challenged groups. Similar results have been reported in human OO, within which the colonization of microbes could induce the accumulation of immune cells known to cause morphological changes in the OO of chronic rhinosinusitis (CRS) patients (26). All of these findings demonstrated that *F. columnare* successfully invaded the trout OO, after which the transcription levels of 15 immune-related genes were detected to evaluate the innate immune responses in the trout OO. These analyses demonstrated that 4 immune genes including CATH-1, IL-10, heat shock proteins, and NOS2 were all significantly upregulated in the OO during early infection. Particularly, cathelicidins contribute substantially to host defense and function as effector molecules of the host innate immune system during bacterial infection (27, 28). These peptides were also upregulated in the nasal mucosa and sinonasal mucosa of chronic rhinosinusitis (CRS) patients, who may suffer from multiple etiologies (*e*.*g*., allergen, bacterial, viral, or fungal exposure) (29, 30). Interleukin-10 (IL-10) is a cytokine with anti-inflammatory properties that limits the immune response to pathogens, thereby preventing damage to the host (31). Additionally, heat shock proteins are not only involved in pro-inflammatory responses but also anti-inflammatory responses (32). NOS-2 mRNA transcription level could be activated within 2–4 h upon LPS-mediated activation of the innate immune pattern recognition receptors (33). Together, our results described the inflammatory responses in trout OO after *F. columnare* infection. Anti-inflammatory and anti-bacterial genes were upregulated in response to *F. columnare* infection, suggesting a tissue repair response. The inflammatory responses of the trout OO increased during *F. columnare* infection; however, the OO also protect itself by limiting the duration of this inflammatory response.

*F. columnare* infection could not only quickly induce a strong innate immune response in the trout OO, but also elicited a long-term adaptive immune response. The mammalian secretory IgA (sIgA) is responsible for the host’s nasal mucosa local defense against bacterial pathogens (15, 34). Given that much higher proportions of IgT^+^ B cells than IgM^+^ B cells were observed in the trout OO (4) and that, similar to the mammalian IgA, IgT is a specialized mucosal Ig isotype against parasitic infection in the gut, gills, and skin of rainbow trout (7, 9, 35), we hypothesized that IgT may also play a key role in teleost NALT against bacterial infection. In order to identify the role of IgT in teleost NALT during bacterial infection, our study was the first to evaluate the immune responses of three Ig isotypes in teleost NALT. The total IgT concentration increased significantly in the nasal mucus of both the infected and surviving fish after *F. columnare* infection, which was consistent with the increased accumulation of IgT^+^ B cells in NALT. Furthermore, the total concentration of another two Igs (IgM and IgD) also increased significantly in nasal mucus after *F. columnare* infection. However, these results are not fully consistent with a previous study, which detected an increased IgT concentration in nasal mucus after parasitic infection, whereas no changes were observed in IgM or IgD concentrations (13). It can be speculated that the differential immune responses of teleost Igs may vary depending on different invading pathogen species. A previous study in humans also detected increased levels of sIgD in nasal tissues but not serum in chronic rhinosinusitis patients after bacterial infection (36). Our study showed that both the protein levels and mRNA expression of IgD significantly increased in nasal mucus after bacterial challenge, whereas serum IgD concentrations exhibited no change, indicating the role of nasal mucosa IgD in the immune responses against *F. columnare* infection. Interestingly, we found differences between protein levels and mRNA expression especially at 28 dpi (i.e., IgT protein levels increased significantly, whereas mRNA expression remained unaltered), which may be due to differences in the mechanisms of action of proteins and mRNA.

We also found that the concentration of IgM in the nasal mucus of infected fish was ~50-fold higher compared to that of IgT, whereas the number of IgT^+^ B cells was ~12-fold higher than that of IgM^+^ B cells in the same fish group. These differences in IgT and IgM concentrations have been observed in all mucosal sites analyzed thus far (7, 9, 13). Moreover, these differences occur not only in mucosal sites but also in serum, where IgM concentrations are ~500-1000 fold higher than those of IgT, whereas the percentage of IgM^+^ B cells in systemic lymphoid organs is only ~3-4 fold higher than that of IgT^+^ B cells. These data indicate that there must be differences in the Ig-secreting capacity of IgM^+^ and IgT^+^ B cells. However, the mechanisms responsible for these variations in Ig-secreting capacities remain largely unknown and therefore should be analyzed in future studies. Interestingly, this phenomenon has also been reported in mammals. For example, the average Ig production by IgA^+^ B cells is significantly different from that of IgM^+^ and IgG^+^ B cells (37). Another possibility that would only apply to mucosal sites is that nasal mucus could have been contaminated with blood during sampling, and therefore we cannot exclude the possibility that the IgM in the nasal mucus derived from blood contamination (i.e., due to potential microhemorrhages that may have occurred during sampling).

Moreover, although the total protein levels of three Ig classes in nasal mucus all increased after *F. columnare* challenge, except for that of bacteria-specific IgM binding could be detected in up to 1/2 nasal mucus dilution in the infected fish, only bacteria-specific IgT titers were detected in the nasal mucus of both the infected and survivor groups. These results were consistent with previous studies in humans in which specific IgA antibodies were detected in nasal mucosa following infection or nasal vaccination, thereby providing protection against *Streptococcus pneumoniae* infection or colonization (15, 38). Although bacteria-specific IgM and IgD levels remained unaltered upon infection, we speculated that increases in total IgM and IgD protein may contribute to the binding of microbes in the OO surface, thereby preventing pathogen invasion. Particularly, *F. columnare* invaded the trout OO, which may have resulted in the colonization of opportunistic pathogens. Importantly, strong IgT and IgM immune responses were detected in teleost serum both at total protein levels and antigen-specific titers after *F. columnare* infection, which was similar to a study in humans that detected increases in antigen-specific IgA and IgG titers in serum after intranasal viral infection (39). These data suggest that IgT may not only function as the main dedicated Ig isotype in nasal immunity but also contributes to systemic immunity during bacterial infection; however, this phenomenon must be further characterized in future studies.

*F. columnare* infection increased the accumulation of IgT^+^ B in trout NALT. However, their origin remains unknown, as they may have been generated locally or transported to the infection site by systemic lymphoid organs. In mammals, most increases in nasal IgA result from the proliferation of local IgA-producing plasma cells in nasal mucosa from patients with chronic inflammation (40). *In vivo* proliferation assays have demonstrated that the percentage of proliferative IgT^+^ B cells also increased significantly in the surviving fish compared to the control fish, indicating that the accumulated IgT^+^ B cells in the nasal mucosa may originate from local proliferation. Furthermore, we also detected high bacteria-specific IgT titers in cultured nasal explants from the surviving fish, which further indicated that the bacteria-specific IgT responses in the OOs could be induced locally during bacterial infection, which was consistent with our previous studies in trout gill and pharyngeal tissues during parasitic infection (9, 24). Overall, our data confirmed that trout NALT is a mucosal inductive for IgT responses, which was similar to the mammalian NALT, where IgA^+^ B cells were induced/expanded and antigen-specific IgA responses were detected following intranasal viral infection (41). Therefore, our results suggest the convergent role of nasal B cells and nasal Ig responses both in tetrapods and non-tetrapods.

In conclusion, our study was the first to explore the Igs specific immune responses of teleost NALTs during bacterial infection. Upon *F. columnare* infection, histopathological changes and innate immune responses were detected in trout NALT, and IgT^+^ B cells were induced and expanded in NALT, which corresponded with increases in bacteria-specific IgT responses in trout NALT and serum. Taken together, our results highlighted IgT as a crucial Ig isotype in trout nasal surface immunity, which may also contribute to systemic immunity. Therefore, our study provides new insights into the nasal adaptive immune responses to bacterial infection and contributes to understand the role of fish NALTs during immersion vaccines.

## Data Availability Statement

The datasets presented in this study can be found in online repositories. The names of the repository/repositories and accession number(s) can be found in the article/**Supplementary Material**.

## Ethics Statement

The animal study was reviewed and approved by Animal procedures were approved by the Animal Experiment Committee of Huazhong Agricultural University.

## Author Contributions

FD and G-mY performed most of the experiments, contributed to the infection model and wrote the manuscript. K-fM contributed to western blot analysis. H-yX and XL performed the immunofluorescence analysis. ZX and Q-cW designed the experiments and revised the manuscript. All authors reviewed the manuscript. All authors contributed to the article and approved the submitted version.

## Funding

This work was supported by grants from the National Natural Science Foundation of China (U1905204, 31873045).

## Conflict of Interest

The authors declare that the research was conducted in the absence of any commercial or financial relationships that could be construed as a potential conflict of interest.
