# Supplementary material for: IgT Plays a Predominant Role in the Antibacterial Immunity of Rainbow Trout Olfactory Organs
Source: Front Immunol. 2020 Nov 16;11:583740. doi: 10.3389/fimmu.2020.583740 (PMC7701277; doi:10.3389/fimmu.2020.583740)
Supplement: Supplementary file 1 [file Presentation_1.pdf]

IgT Plays a Predominant Role in the Antibacterial Immunity of Rainbow Trout Olfactory Organs

Fen Dong[1,†], Guang-mei Yin[1,†], Kai-feng Meng[1], Hao-yue Xu[1], Xia Liu[1], Qing-chao Wang[1], Zhen Xu[1,2,3] *

[1] Department of Aquatic Animal Medicine, College of Fisheries, Huazhong Agricultural University, Wuhan, Hubei, 430070, China;

[2] Laboratory for Marine Biology and Biotechnology, Qingdao National Laboratory for Marine Science and Technology, Qingdao, Shandong 266071, China;

[3] Key Laboratory of Marine Biotechnology of Fujian Province, Institute of Oceanology, Fujian Agriculture and Forestry University, Fuzhou, 35002, China.

*Corresponding author, *E-mail*: zhenxu@mail.hzau.edu.cn.

[†]These Authors contributed equally to this paper.

A

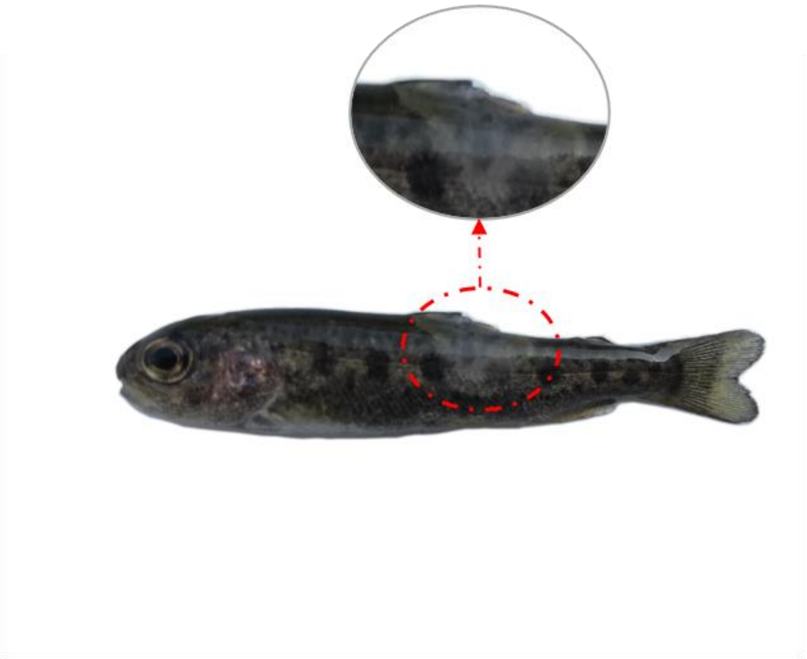

B

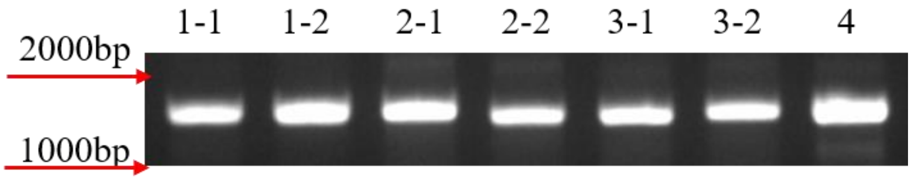

FIGURE S1 | Successful *F. columnare* infection in olfactory organ of trout. **(A)** The classical phenotype of columnaris disease, showed skin were pale color and fin rot. **(B)** Detection of single bacterial colonies in plate A from Figure 1A by PCR (right; the red arrows point out three different bacteria colony). The positive control of *F. columnare* (4).

A

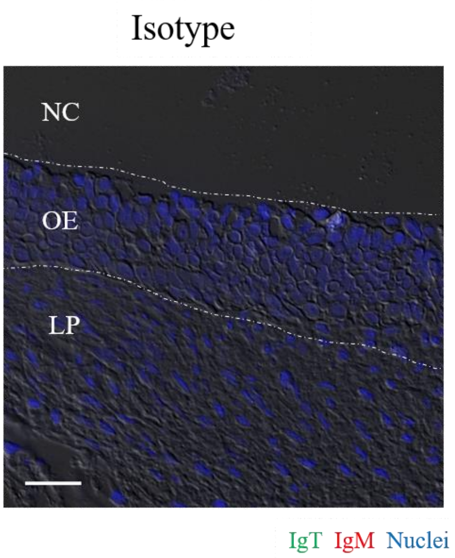

FIGURE S2 | Isotype control staining for anti-IgT and anti-IgM antibodies in trout olfactory organ paraffin-sections. DIC images of olfactory organ paraffin-sections from control fish, with merged staining of isotype control antibodies for anti-trout IgT pAb (green) and anti-trout IgM (red) mAb. Nuclei were stained with DAPI (blue). NC, nasal cavity; OE, olfactory epithelium; LP, lamina propria. Scale bars, 20 μm. Data are representative of three independent experiments.

FIGURE 3E

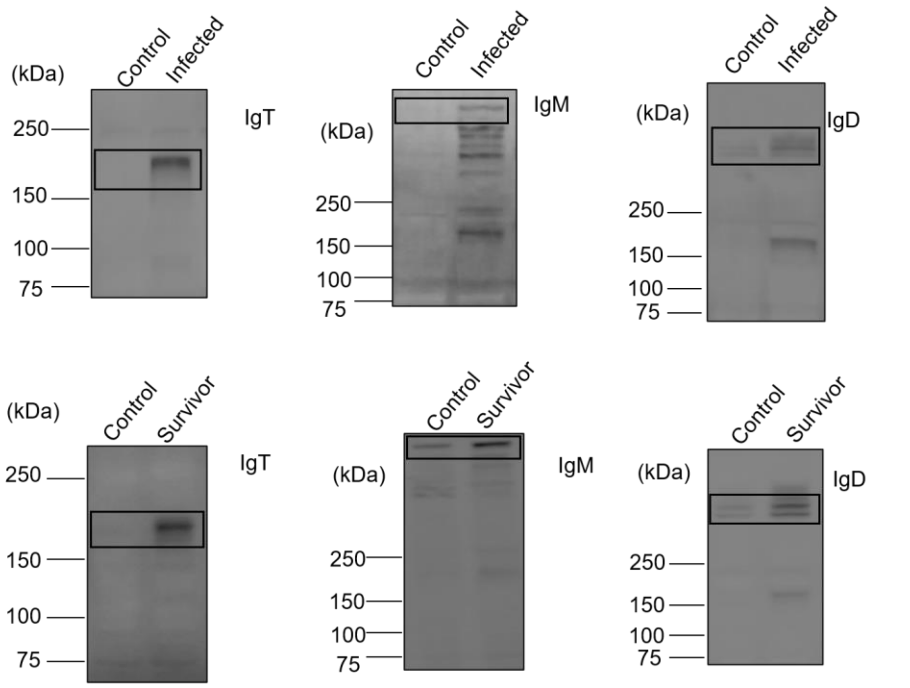

FIGURE 3F

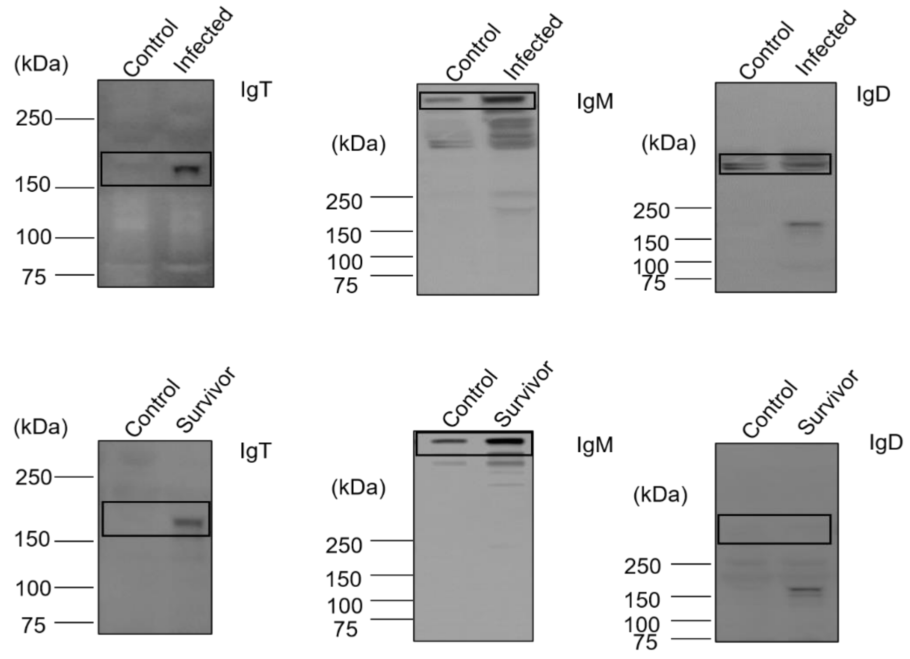

FIGURE S3 | Original images of the western blot analyses in the main figures.

FIGURE 4A

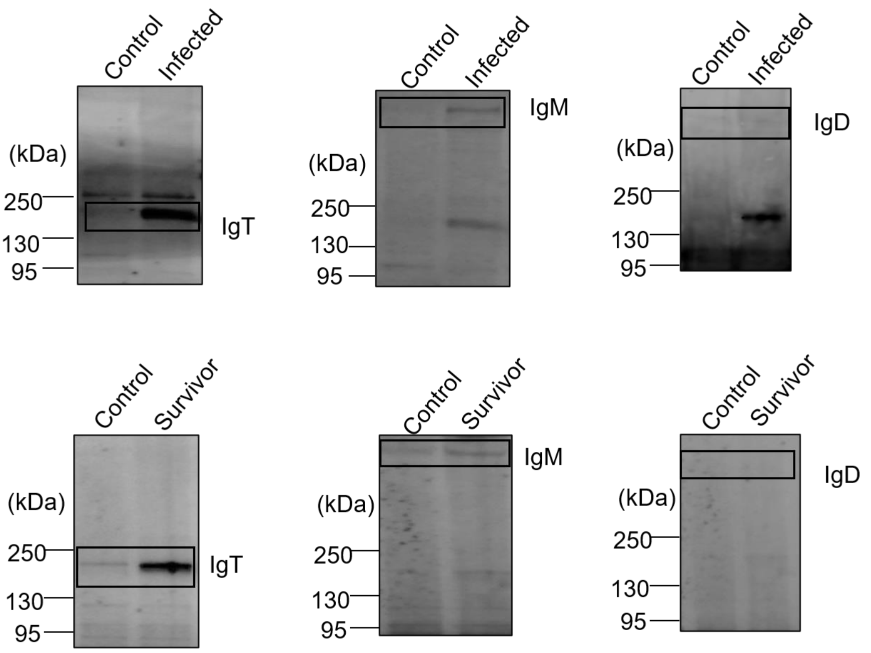

FIGURE 4D

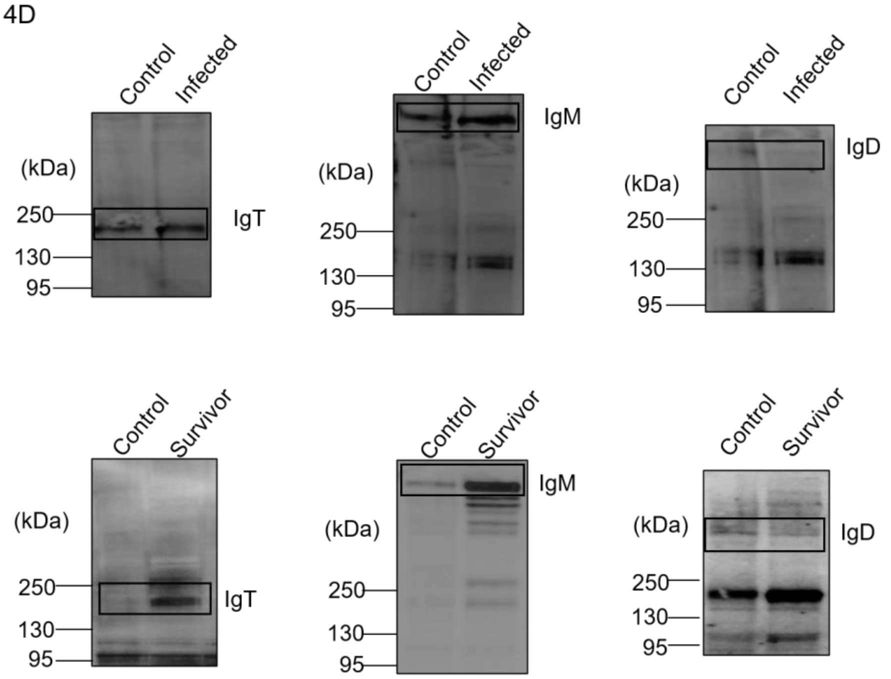

FIGURE 6A

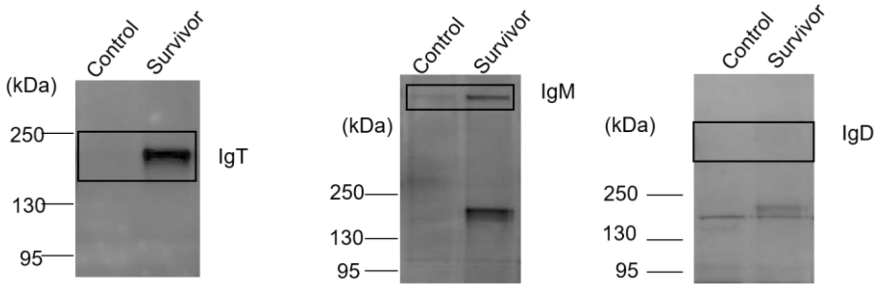

FIGURE S4 | Original images of the western blot analyses in the main figures.
